# Small Intestine Inflammation in Roquin-Mutant and Roquin-Deficient Mice

**DOI:** 10.1371/journal.pone.0056436

**Published:** 2013-02-25

**Authors:** Jeremy S. Schaefer, Dina Montufar-Solis, Niyati Nakra, Nadarajah Vigneswaran, John R. Klein

**Affiliations:** Department of Diagnostic and Biomedical Sciences, University of Texas Health Science Center School of Dentistry, Houston, Texas, United States of America; Charité-University Medicine Berlin, Germany

## Abstract

Roquin, an E3 ubiquitin ligase that localizes to cytosolic RNA granules, is involved in regulating mRNA stability and translation. Mice that have a M199R mutation in the Roquin protein (referred to as sanroque or *Roquin^san/san^* mice) develop autoimmune pathologies, although the extent to which these occur in the intestinal mucosa has not been determined. Here, we demonstrate that *Roquin^san/san^* mice reproducibly develop intestinal inflammation in the small intestine but not the colon. Similarly, mice generated in our laboratory in which the *Roquin* gene was disrupted by insertion of a gene trap cassette (*Roquin^gt/gt^* mice) had small intestinal inflammation that mimicked that of *Roquin^san/san^* mice. MLN cells in *Roquin^san/san^* mice consisted of activated proliferating T cells, and had increased numbers of CD44^hi^ CD62L^lo^ KLRG1^+^ short-lived effector cells. Proportionally more small intestinal intraepithelial lymphocytes in *Roquin^san/san^* mice expressed the ICOS T cell activation marker. Of particular interest, small intestinal lamina propria lymphocytes in *Roquin^san/san^* mice consisted of a high proportion of Gr-1^+^ T cells that included IL-17A^+^ cells and CD8^+^ IFN-γ^+^ cells. Extensive cytokine dysregulation resulting in both over-expression and under-expression of chemotactic cytokines occurred in the ileum of *Roquin^san/san^* mice, the region most prone to the development of inflammation. These findings demonstrate that chronic inflammation ensues in the intestine following Roquin alteration either as a consequence of protein mutation or gene disruption, and they have implications for understanding how small intestinal inflammation is perpetuated in Crohn's disease (CD). Due to the paucity of animal models of CD-like pathophysiology in the small intestine, and because the primary gene/protein defects of the Roquin animal systems used here are well-defined, it will be possible to further elucidate the underlying genetic and molecular mechanisms that drive the disease process.

## Introduction

Roquin, a RING finger E3 ubiquitin ligase that is characterized by a CCCH zinc finger found in RNA-binding proteins, localizes to cytosolic RNA granules that are involved in regulating mRNA stability and translation. As a member of the ubiquitin-mediated protein degradation pathway, E3 ligases determine the specificity of degradation by associating with a substrate [1]. E3 ligases are further defined by their catalytic domains: Homologous to E6-AP Carboxyl Terminus (HECT) domain proteins have intrinsic ligase activities, whereas Really Interesting New Gene (RING) domain proteins function as scaffolds in the degradation process [2]. Initial characterization of Roquin-defective mice (*Roquin^san/san^*), which have an M199R mutation in the Roquin protein, revealed chronic inflammation consisting of lymphadenopathy, splenomegaly, thrombocytopenia, necrotizing hepatitis, and increased numbers of CD4^+^ follicular T cells and ICOS expression [3], [4]. A recent study from our laboratory demonstrated that Roquin curtails IL-17 synthesis, and that the miR-223 microRNA is involved in regulating Roquin expression [5].

The extent to which Roquin is involved in driving autoimmunity has been brought into question, however, by a recent study using complete and conditional *Roquin* knockout mice [6]. Complete *Roquin* knockout resulted in a deformity of the caudal spine, and was generally lethal for most mice within 6 hours of birth, possibly due to a defect in lung development. Conditional knockouts in which the *Roquin* defect was targeted to T cells failed to exhibit a breach in self-tolerance or have changes in follicular T cell differentiation, although increased ICOS expression was noted [6]. Similar findings were observed for mice in which *Roquin* disruption was targeted to the entire hematopoietic system [6]. The reason for the discrepancy between findings from *Roquin^san/san^* and *Roquin*-deficient mice remains unclear; however, it is possible that a mutated form of the Roquin protein rather than total ablation of it is a contributing factor in promoting autoimmunity.

Based on the above observations, we were interested in determining the extent to which *Roquin^san/san^* mice, or mice in which the *Roquin* gene has been disrupted, develop intestinal inflammation. For the latter, we generated mice in which a gene trap was inserted into the *Roquin* gene (*Roquin^gt/gt^* mice). Here, we report that inflammation throughout the small intestine, but not in the colon, is a common feature of both *Roquin^san/san^* and *Roquin^gt/gt^* mice. Additionally, the ileum of *Roquin^san/san^* mice, the portion of the small intestine most prone to development of inflammation, had extensive cytokine dysregulation, activated T cells in mesenteric lymph nodes (MLN), small intestinal intraepithelial lymphocytes (siIELs) and small intestinal lamina propria lymphocytes (siLPLs), and pro-inflammatory cytokine production by siLPLs. These findings demonstrate alteration in the Roquin protein, either as a consequence of mutation or gene disruption, is a causal factor tied to the development of chronic inflammation in the small intestine.

## Materials and Methods

### Ethics Statement

All experiments were performed in accordance with the National Institutes of Health Guide for the Care and Use of Laboratory Animals using a protocol approved by the University of Texas Health Science Center Institutional Animal Welfare Committee (Permit Number: HSC-AWC-12-039).

### Mice

Mating pairs of *Roquin^san/+^* mice were obtained from the Mutant Mouse Regional Resource Center (MMRRC) (University of California, Davis, CA) to establish a colony in the animal facility at The University of Texas Health Science Center at Houston. Heterozygous *Roquin^san/+^* mice were intercrossed to produce homozygous *Roquin^san/san^* mice. A mutagenically-separated PCR (MS-PCR) protocol [3] was used to determine zygosity. A PCR product of approximately 190 bp indicated the wild type allele while a product of approximately 215 bp denoted the presence of the *Roquin^san^* mutation. Heterozygous mice displayed both products. Primers were: San forward, 5′-CATCAGGCTGGCCTCTAGTT-3′; San reverse M199, 5′- AAGACCAGCTTCAGAGCTTCCTCCAGCA-3′; San reverseR199, 5′-GAACCATCTTCTAAGGCCAGCGTGACCAGCTTCAGAGCTTCCTCCTACC-3′.

To generate *Roquin* gene trap mice, the SIGTR CC0753 ES cell line targeting the *Roquin* gene was purchased from the Mutant Mouse Regional Resource Center (MMRRC) (University of California, Davis, CA). Initial characterization of the ES cell line via Blasting of the sequence tag localized the gene trap within intron 1 of *Rc3h1*. In order to determine the precise location of the gene trap, we designed sequential PCR primers to amplify 2–4 kb regions between exon 1 and exon 2 in *Rc3h1*. PCR amplification using these *Rc3h1* specific primers coupled to the reverse β-geo specific primer revealed the location of the gene trap insertion to be between P5099 and P6546 of intron 1. The PCR product resulting from the amplification using the Rc3h1 P5099 and β-geo reverse primers was TOPO cloned and sequenced. Sequencing analysis identified the gene trap insertion point as following base pair 5809 within intron 1 of *Rc3h1* (Fig. S1).

The ES cell clones were expanded and microinjected into blastocysts to generate the gene trap chimeras at the Laboratory for Developmental Biology at The Brown Foundation Institute of Molecular Medicine (The University of Texas Health Science Center at Houston, Houston, TX). Chimeric mice were screened for the presence of the β-geo gene trap cassette using gene trap-specific primers. Gene trap-positive chimeras were backcrossed to the C57BL/6 background for more than eight generations. *Roquin^gt/+^* heterozygous mice were intercrossed to obtain homozygous *Roquin^gt/gt^* mice. PCR analysis using Roquin-specific primers that span the gene trap insertion point was performed to establish zygosity. The gene trap and Roquin-specific primers were: β-geo forward, 5′-CCCAACAGTTGCGCAGCCTGAAT-3′; β-geo reverse, 5′- CGCTGCACCATTCGCGTTAC-3′; Roquin P5634 forward, 5′-AGGCCAGCCTGGTGAAATAG-3′; Roquin P6120 reverse, 5′-AGGCCAGCCCAGTCTACAG-3′. *Roquin^gt/gt^* mice had a poor post-birth survival rate and most had a congenital caudal spine defect. Out of 29 litters obtained from heterozygous crosses, the ratio was 1.57 [wild type]: 4.3: [heterozygous]: 0.12 [homozygous gene trap] compared to the predicted simple Mendelian ratio of 1∶2∶1.

To measure Roquin and ICOS transcript levels, a Power SYBR Green PCR Master Mix kit (Applied Biosystems) was used according to the manufacturer's instructions. Total RNA was isolated using an miRNeasy Minikit (Qiagen, Valencia, CA) according to the manufacturer's instructions. cDNA was synthesized using a High-Capacity cDNA Reverse Transcription Kit (Applied Biosystems, Austin, TX). Samples were analyzed using the StepOnePlus real-time thermal cycler and software (Applied Biosystems). Relative gene expression was normalized to GAPDH. Roquin, IL-17A, and GAPDH gene-specific primers were designed and purchased from Integrated DNA Technologies (Coralville, IA). Primers were: Roquin forward, 5′-GGCTGCTCGATCTTTAGGTG-3′; Roquin reverse, 5′-TGTTCTCTCCTCAGAGCTTCG-3′; ICOS forward, 5′-TGACCCACCTCCTTTTCAAG-3′; ICOS reverse, 5′-TTAGGGTCATGCACACTGGA-3′; GAPDH forward, 5′-AGAACATCATCCCTGCATCC-3′; GAPDH reverse, 5′-AGCCGTATTCATTGTCATACC-3′.

Roquin western blotting was done using spleen tissues or cultured cells that had been lysed and homogenized in RIPA buffer (50 mM Tris-cl pH 7.4, 150 mM NaCl, 1% NP40, 1% SDS, 0.5% Na-deoxycholate, 1 mM PMSF, 50 mM NaF, aprotinin, leupeptin, pepstatin: 1 µg/ml each). 40 µg of cell lysates were electrophoresed through an 8% SDS polyacrylamide gel followed by overnight protein transfer to PVDF membranes. Membranes were blocked using 5% milk in TBS containing 0.1% Tween-20 (TBS-T_0.1_). Membranes were probed with a Roquin antibody (Bethyl Laboratories, Montgomery, TX; A300-514A, [1∶560]) overnight at 4°C with rocking, washed x3 with TBS-T_0.1_, and incubated with an HRP-conjugated goat anti-rabbit antibody (Vector Laboratories, Burlingame, CA; [1∶1500]). Enhanced chemiluminescent (SuperSignal West Pico Chemiluminescent Substrate [Thermo Fisher Scientific, Rockford, IL]) was used to detect the protein bands.

Adult female C57BL/6 mice, 8–10 weeks of age, were purchased from Harlan (Indianapolis, IN) and used for MLN, siIEL, and siLPL cell isolations, and for breeding with *Roquin^gt/+^* mice.

### Histopathological and morphometric analysis

Mice were euthanized, intestinal tissues from the duodenum, jejunum, ileum, and cecum, and the ascending, transverse, and descending, colon, and the liver were harvested, fixed in 10% buffered formalin, processed for paraffin embedding, and 3 µM tissue sections were made and stained with hematoxylin and eosin (H&E). The degree of inflammation and related histologic changes were graded microscopically on cross-sections of intestinal tissues using a validated scoring system as described in our previously published paper [7]. Scoring for intestinal tissues consisted of score 0: no signs of inflammation; score 1: very low level of leukocytic infiltration in the lamina propria; score 2: low level of leukocytic infiltration in the lamina propria; score 3: moderate level of leukocytic infiltrate in the lamina propria with occasional crypt abscess; score 4: high levels of leukocytic in the lamina propria, crypt abscess, high vascular density, loss of goblet cells, and thickening of the intestinal wall; and score 5: transmural leukocytic infiltration, loss of goblet cells, high vascular density, and thickening of the intestinal wall. Scoring for the liver was done as described by others [8]. An experienced, board-certified pathologist performed histopathologic grading in a blinded manner.

Body weight of female *Roquin^san/san^* and C57BL/6 mice were recorded between 14 and 55 weeks of age. After euthanasia, spleen-to-body weight ratios were calculated from the body and spleen weights.

### Leukocyte isolations and flow cytometric analyses

T cells were isolated from MLNs using a Mouse T-Cell Enrichment Kit (R&D, Minneapolis, MN) according to the manufacturer's protocol. siIELs were isolated using a protocol previously reported by our laboratory [9]. For isolation of siLPLs, the epithelial layer was first digested in DTT/EDTA [9], removed, and the remaining intestinal fragments were incubated in 50 ml of RPMI supplemented with 10% FBS, 0.36 mM CaCl_2_, 100U/ml of Collagenase IV (Sigma, St. Louis, MO) for 1 h at 37°C with stirring. Cells were filtered through nylon wool, collected, and centrifuged at 400 g through a 40%/70% Percoll gradient. Viable cells were collected from the interface.

MLN cells, siIELs, and siLPLs were stained for identification of surface or intracellular markers as previously described [7] using combinations of the following antibodies: PE-anti-CD134 (OX40) (OX-86); PE-anti-CD278 (ICOS) (7E.17G9); PE-anti-FoxP3 (FJK-16 s); APC-anti-CD3ε (145-2C11); FITC-anti-CD44 (IM7); APC-anti-CD25 (PC61.5); AlexaFluor647-anti-Ki67 (SolA15); APC-anti-KLRG1 (2F1); APC- and PE-anti-IL-17A (17B7); FITC-anti-IFNγ (XMG1.2); PE-anti-CD8a (53–6.7); FITC rat IgG isotype control (eBRG1); biotin rat IgG isotype control; PE rat IgG2a isotype control (eBR2a); streptavidin APC (eBioscience, San Diego, CA, all reagents); FITC anti-mouse CD45R/B220 (RA3-6B2); PE-anti-CD3ε (145-2C11); FITC-anti-BrdU (3D4); PE-anti-CD62L (MEL-17) (Pharmingen, San Diego, CA, all reagents); biotin-anti Ly6C (HK1.4) (Southern biotechnology, Birmingham, AL). BrdU was administered by an i.p. injection of 1 mg BrdU at 72 and 48 hours prior to cell isolation.

### Analyte arrays

Tissues from the ileum of normal and *Roquin^san/san^* mice were collected. Half was stored in RNAlater (Qiagen, Valencia, CA), half was used for histopathological analyses. RNA was isolated from tissues of three normal mice and three mice with pathology scores of 1.0. A RT^2^ Profiler PCR Array Mouse Inflammatory Cytokines & Receptors kit (Qiagen) was used to measure the expression of 89 analytes consisting of genes for 28 chemokines, 13 chemokine receptors, 25 cytokines, 14 cytokine receptors, and 9 additional inflammation-related genes according to the manufacturer's protocols.

### Statistical analysis

Determination of statistical significance was done using an unpaired two-sided Student's t-test.

## Results

### *Roquin^san/san^* and *Roquin^gt/gt^* mice develop inflammation in the small intestine and liver

**Figure 1:**
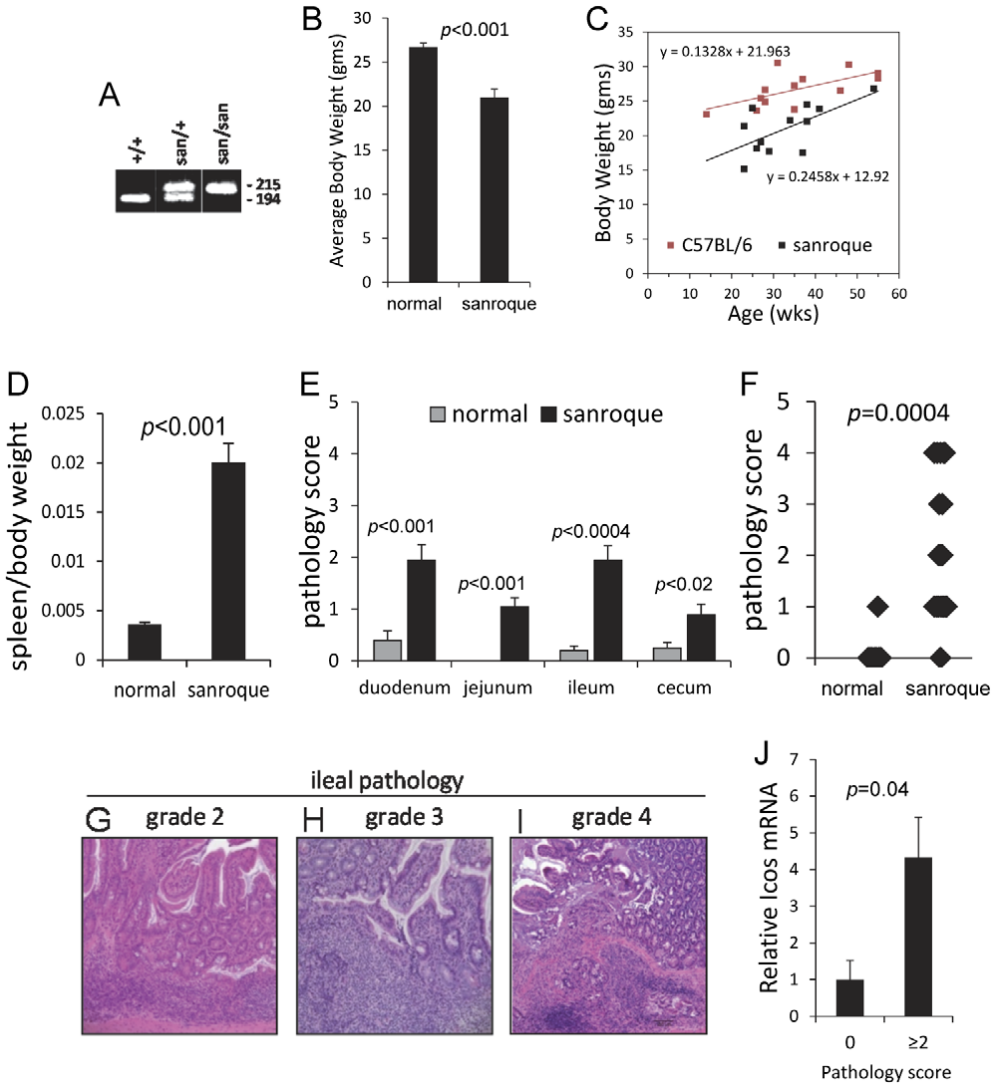
Morphometric and intestinal histopathological features of *Roquin^san/san^* mice. (**A**) MS-PCR was used to identify homozygous *Roquin^san/san^* mutant as demonstrated by the presence of a single 215 bp PCR product. Heterozygous *Roquin^san/san^* mice had two PCR products. *Roquin^san/san^* mice employed in the study were confirmed by MS-PCR. (**B, C**) Body weight of *Roquin^san/san^* mice 15–55 weeks of age was significantly less than that of gender-matched normal animals. Mean ± SEM of 13 normal and 12 *Roquin^san/san^* mice. (**D**) *Roquin^san/san^* mice also had pronounced splenomegaly with follicular lymphoid hyperplasia, a characteristic of this animal model. Mean ± SEM of 7 normal and 12 *Roquin^san/san^* mice ages 15–55 weeks. (**E**) Histopathological analysis of intestinal tissues revealed inflammation throughout the small intestine and cecum. Mean ± SEM of pathology scores of tissues from 23 *Roquin^san/san^* mice and 8–10 normal mice ages 15–55 weeks. (**F**) Distribution of pathology scores for the ileum of *Roquin^san/san^* mice as shown in panel E; note that only 2 of 23 *Roquin^san/san^* mice had no inflammation or mucosal injury in the ileum. (**G–I**) Representative H&E stained tissue sections from the ileum of *Roquin^san/san^* mice with pathology scores of 2, 3, and 4 that were characterized by villous atrophy and ulceration, crypt hyperplasia and increased intraepithelial mononuclear cell infiltrate. Original magnification, 200x. (**J**) *Icos* gene expression was elevated in the ileum of *Roquin^san/san^* mice. Mean ± SEM of three replicate samples ages 16, 17, and 26 weeks.

We confirmed *Roquin^san/san^* mice to be homozygous mutant animals by MS-PCR [3]. As shown in Fig. 1A, normal mice had a single 190 bp PCR product. Homozygous *Roquin^san/san^* mutant mice had a single 215 bp PCR product. Heterozygous *Roquin* ^san/+^ mice had both PCR products. The average body weight of *Roquin^san/san^* mice from 14 to 55 weeks of age was significantly less than that of gender-matched normal animals (Fig. 1B), although with age the weight of *Roquin^san/san^* mice more closely approximated that of normal animals (Fig. 1C). Pathology scores did not show a significant positive or negative correlation with age (not shown). *Roquin^san/san^* mice had pronounced splenomegaly (Fig. 1D) that was characterized by florid follicular lymphoid hyperplasia (not shown). Histopathological analysis of intestinal tissues from *Roquin^san/san^* mice revealed inflammation throughout the small intestine and cecum with varying degrees of chronic inflammation spread along the entire length of the small intestine that was characterized by elongated crypts with flattened villi and increased siIELs. In focal areas, the intestinal inflammation was associated with ulceration of the villi, crypt abscess, and inflammatory cell infiltrates extending into the lamina propria. The pathology scores for the small intestine are shown in Fig. 1E. The distribution of pathology scores for the ileum of 23 *Roquin^san/san^* mice is displayed in Fig. 1F. Only 2 of 23 *Roquin^san/san^* mice had no pathology in the ileum. Tissue sections from the ileum of *Roquin^san/san^* mice with pathology scores of 2, 3, and 4 are shown in Fig. 1G–I, respectively. Inflammation in the colon of *Roquin^san/san^* mice was rare (not shown). Similar to other studies in which ICOS expression was elevated in splenic CD4^+^ T cells of *Roquin^san/san^* mice [4], *Icos* gene expression was elevated in the ileum (Fig. 1J).

**Figure 2:**
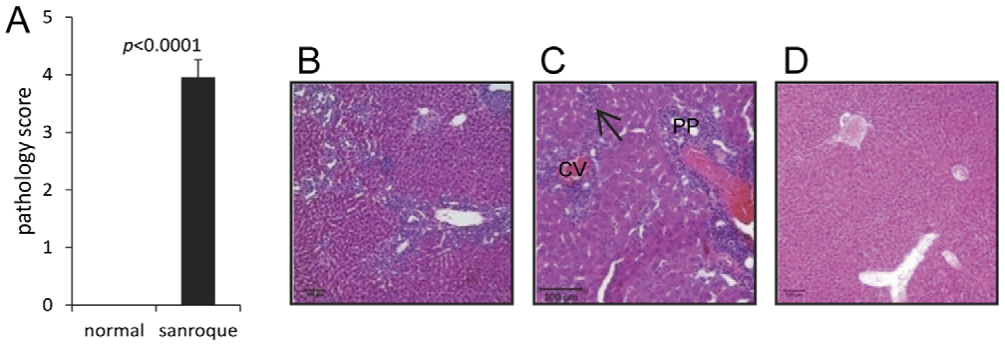
*Roquin^san/san^* mice have chronic hepatitis. (**A**) Average pathology score of normal and *Roquin^san/san^* mice. Mean ± SEM of 6 normal and 26 *Roquin^san/san^* mice ages 15–55 weeks. (**B, C**) Representative H&E stained tissue sections of the liver from *Roquin^san/san^* mice with pathology scores of 5. (**C**) Mononuclear cell infiltrates are seen distributed in the periportal area (PP) and around the central veins (CV) of the hepatic lobules. Focal areas of hepatocyte necrosis (arrows) with mononuclear cell infiltration is found in the mid-zonal and pericentral areas of the hepatic lobules. (**D**) A representative H&E stained liver section from a normal C57BL/6 mouse devoid of inflammation. Original magnifications, B and D, 100×; C, 200×.

The liver of 26 *Roquin^san/san^* mice revealed moderate-to-severe hepatitis with an average pathology score of 3.96±0.29 (Fig. 2A). The degree of inflammation and liver cell death were graded with minor modification using a published scoring system for necroinflammatory activity [8]. Hematoxylin and eosin stained sections of *Roquin^san/san^* mice liver showed varying degrees of mononuclear cell infiltrate around central vein and periportal areas associated with lobular hepatocyte necrosis (Fig. 2C). Occasionally, chronic granulomatous inflammation consisting of lymphocytes, histiocytes and multinucleated giant cells were also noted around the central vein. The histopathological features of the liver from *Roquin^san/san^* mice with pathology scores of 5 are shown in Fig. 2B and C. A representative liver section from a normal animal without inflammation or hepatocyte death is shown (Fig. 2D).

**Figure 3:**
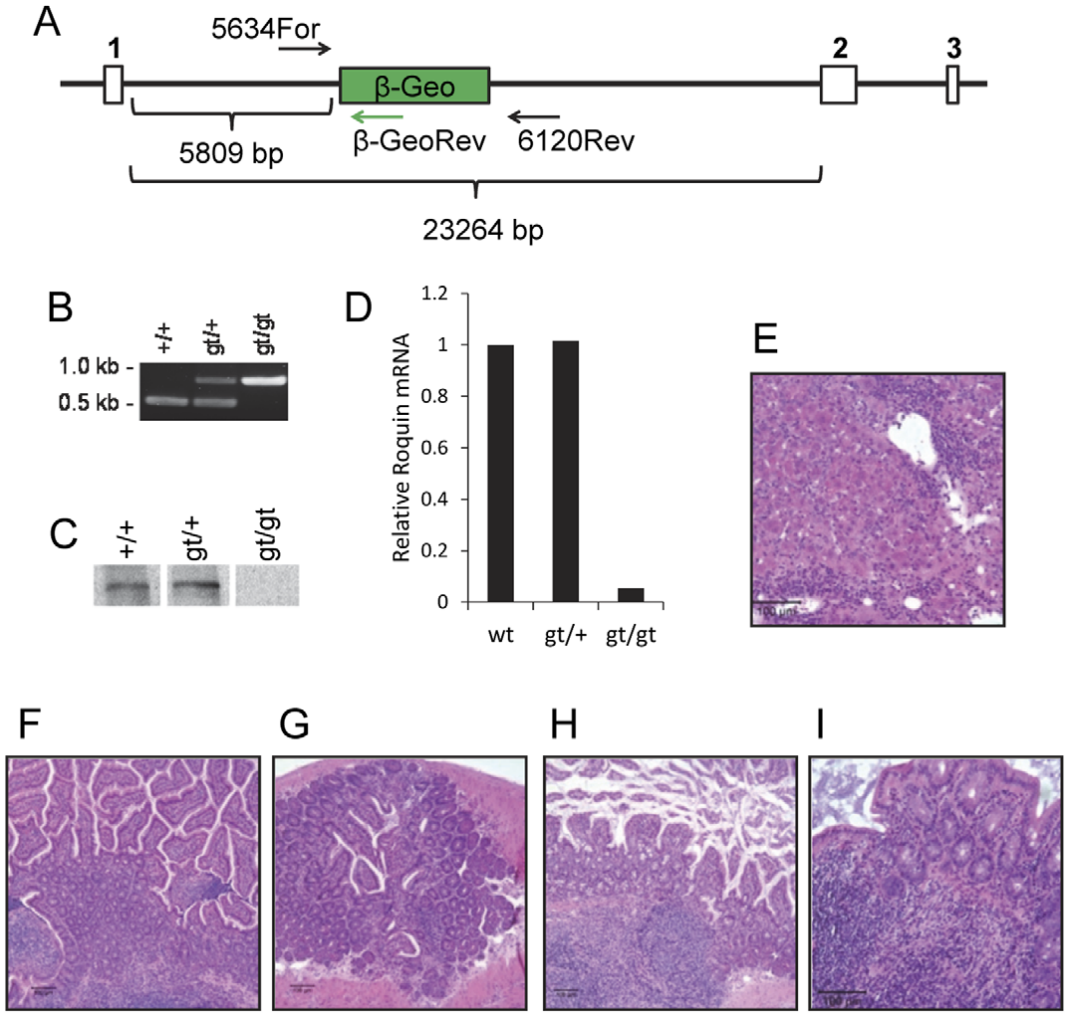
*Roquin^gt/gt^* gene trap mice develop extensive small intestine and liver inflammation. (**A**) The location of the β-geo gene trap cassette and genotyping primers relative to the *Roquin* exons. (**B**) *Roquin^gt/gt^* mice were screened using gene trap-specific primers. Mice homozygous for the gene trap had a single band of 800 bp; heterozygotes had two bands of 800 bp and 486 bp; wild type mice had a single 486 bp band. Animal ages 16–22 weeks. (**C**) The Roquin protein was undetectable in the spleen of homozygous (gt/gt) mice by western blotting; 1animal each. Animal ages 16–22 weeks. (**D**) Roquin gene expression in the intestine of homozygous (gt/gt) mice was reduced by ∼95%; 1animal each. Animal ages 16–22 weeks. Representative H&E stained tissue sections of the (**E**) liver, (**F**) duodenum, (**G**) jejunum, (**H**) ileum, and (**I**) cecum of a *Roquin^gt/gt^* mouse exhibited chronic hepatitis and intestinal inflammation. Pathology scores of sections are described in the text. E–I, original magnifications, 200×.

To further understand the involvement of Roquin in modulating the inflammatory response, we generated Roquin-deficient mice using a Sanger Institute Gene Trap ES cell line (SIGTR CC0753) that targeted the *Roquin* gene (*Roquin^gt/gt^*). Fig. 3A illustrates the location of the β-geo gene trap cassette and genotyping primers relative to the *Roquin* exons. Mice were bred onto the C57BL/6 background for more than eight generations. Mice homozygous for the gene trap had a single band of 800 bp; heterozygotes had two bands of 800 bp and 486 bp; wild type mice had a single 486 bp band (Fig. 3B). Roquin gene expression was reduced by 95% in the intestine compared to wild type or heterozygous animals (Fig. 3D). Importantly, however, the Roquin protein was undetectable in the spleen of *Roquin^gt/gt^* mice by western blotting (Fig. 3C). Tissue sections from the liver (grade 3), duodenum (grade 2), jejunum (grade 2), ileum (grade 2), and cecum (grade 3) of a *Roquin^gt/gt^* mouse are shown in Fig. 3E–I, respectively. Microscopic features of intestinal inflammation and chronic hepatitis in *Roquin^gt/gt^* mice were very similar to that of *Roquin^san/san^* mice. The average pathology score in the small intestine of three *Roquin^gt/gt^* mice was: 1.67±0.75 for the duodenum, 2.00±0.83 for the jejunum, 2.66±0.33 for the ileum, and 1.66±0.75 for the cecum. As with *Roquin^san/san^* mice, the most severe inflammation in *Roquin^gt/gt^* mice occurred in the ileum. All three *Roquin^gt/gt^* mice lacked colonic inflammation (not shown).

### MLN cells in *Roquin^san/san^* mice have increased numbers of activated T cells, a higher proportion of proliferating T cells, and more short-lived effector cells (SLECs)

**Figure 4:**
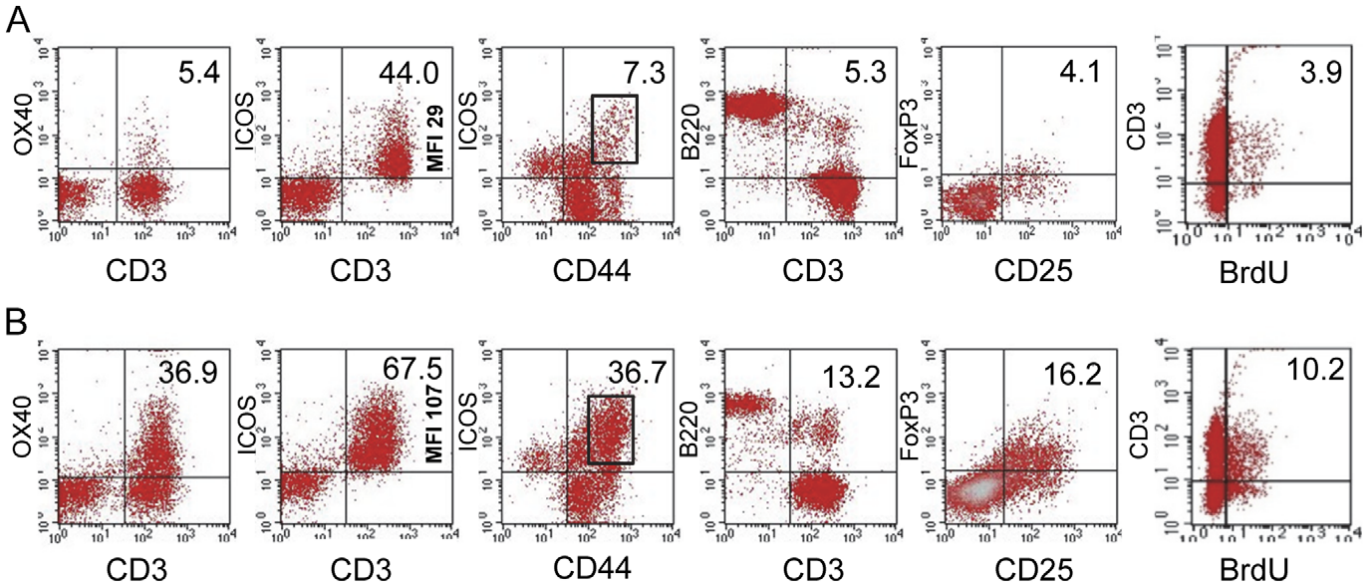
MLN cells in *Roquin^san/san^* mice have increased numbers of activated T cells, proliferating cells, and regulatory T cells. MLN lymphocytes from (**A**) normal and (**B**) *Roquin^san/san^* mice based on expression of OX40, ICOS, B220, FoxP3, and BrdU incorporation. FoxP3 CD25 expression was determined after gating onto CD4^+^ cells. Representative data from 2–3 normal and 2–4 *Roquin^san/san^* mice.

**Figure 5:**
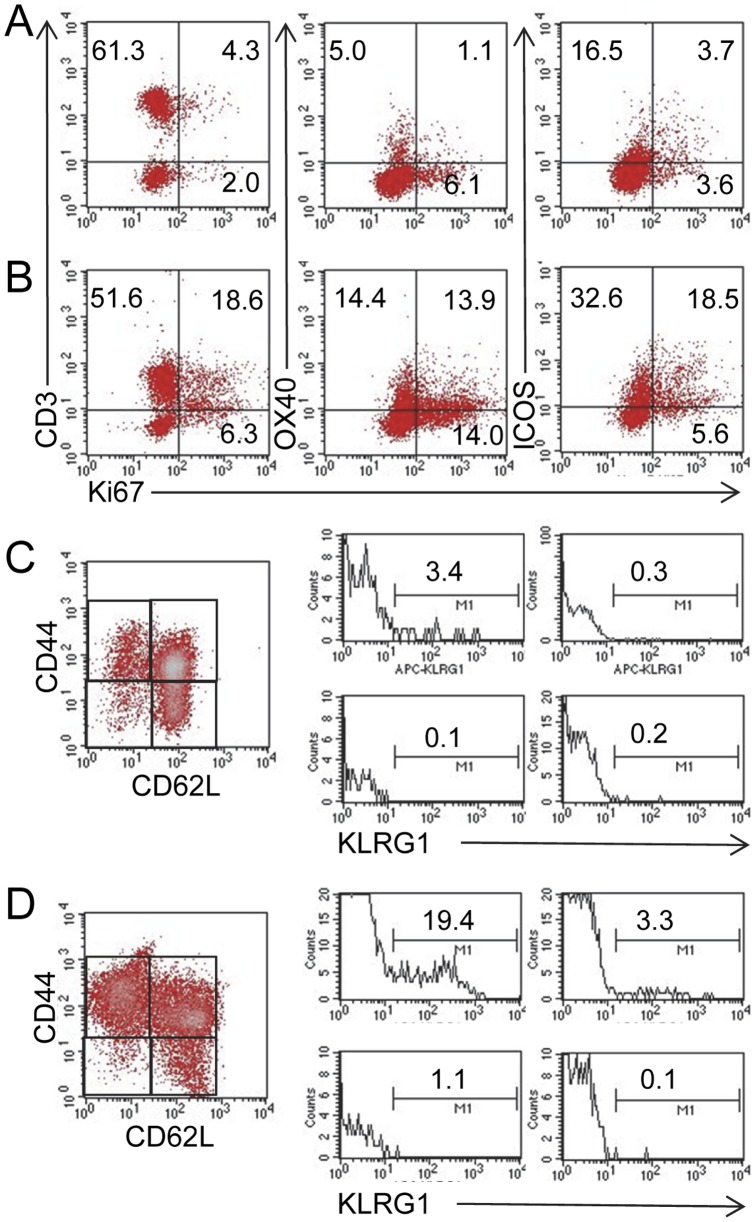
MLN cells from *Roquin^san/san^* mice are proliferative and have more SLECs. Based on Ki67 staining, (panel **B**) a greater proportion of OX40^+^ cells and ICOS^+^ MLN cells were proliferating T cells compared to MLN cells from (panel **A**) normal mice. Representative staining from 1 normal and 2 *Roquin^san/san^* mice. Similarly, there was a greater proportion of CD44^hi^ CD62L^lo^ KLRG1^+^ SLECs present in MLN cells of (panel **D**) *Roquin^san/san^* mice compared to (panel **C**) normal mice. Representative data from 3 normal and 3 *Roquin^san/san^* mice.

Compared to normal C57BL/6 mice (Fig. 4A), MLN T cells from *Roquin^san/san^* mice (Fig. 4B) had proportionally more CD3^+^ OX40^+^, CD3^+^ ICOS^+^, ICOS^+^ CD44^hi^, and CD3^+^ B220^+^ cells. Additionally, ICOS was expressed at a higher MFI in *Roquin^san/san^* mice than normal mice (107 vs. 29, respectively). CD25^+^ FoxP3^+^ Tregs (gated onto CD4^+^ cells) were more abundant in *Roquin^san/san^* mice, and CD3^+^ T cells were more proliferative based on in vivo BrdU incorporation (Fig. 4). The elevated proliferative response of OX40^+^ and ICOS^+^ cells was further confirmed by ex vivo Ki67 staining (Fig. 5A and B). An additional important finding was the greater number of SLECs in the MLN of *Roquin^san/san^* mice (Fig. 5D) compared to normal mice (Fig. 5C) as defined by CD44^hi^ CD62L^lo^ KLRG1^+^ expression.

### Small intestine IELs and LPLs in *Roquin^san/san^* mice have unique phenotypic profiles linked to inflammation

**Figure 6:**
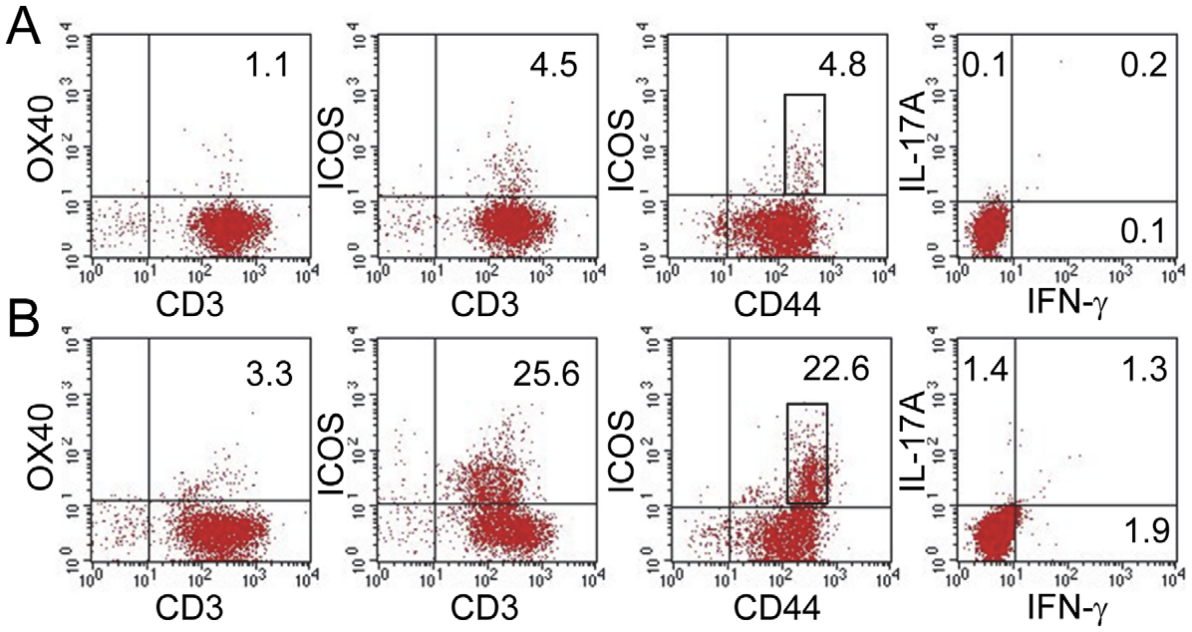
Characterization of siIELs in *Roquin^san/san^* mice. siIELs from *Roquin^san/san^* mice have more (panel **B**) ICOS^+^ T cells, including a subset of CD44^hi^ ICOS^+^ cells compared to (panel **A**) normal animals. Interestingly, neither IL-17A nor IFN-γ were produced to any appreciable levels in either type of mouse (panels A and B). Representative data from 2 normal and 2 *Roquin^san/san^* mice.

We characterized siIELs and siLPLs from *Roquin^san/san^* mice according to expression of function-associated activation markers and pro-inflammatory cytokines. Findings from these studies revealed differences in the activation status of cells depending upon the mucosal lymphoid cell compartmentalization. Unlike MLN cells of *Roquin^san/san^* mice, few siIELs expressed OX40. ICOS was expressed on approximately one-quarter of all CD3^+^ and CD44^hi^ siIELs from *Roquin^san/san^* mice (Fig. 6B). IL-17A and IFN-γ were not produced to any appreciable level by either *Roquin^san/san^* or normal mice (Fig. 6A and B).

**Figure 7:**
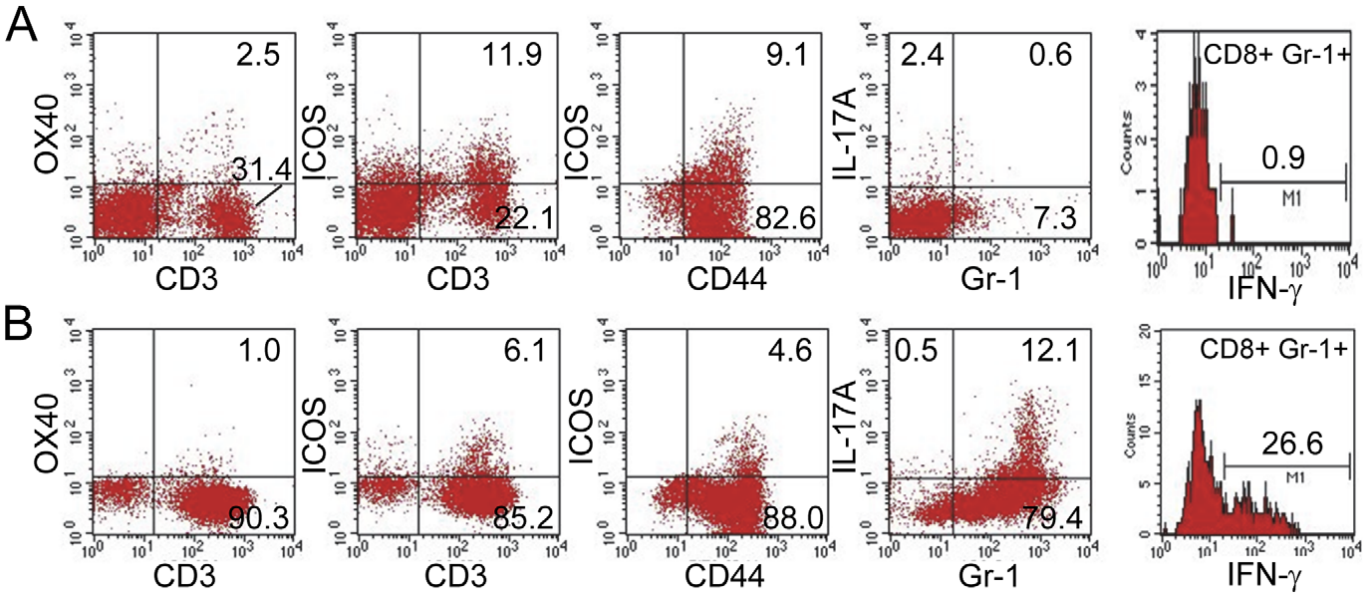
Unique phenotypic profile of siLPLs in *Roquin^san/san^* mice. siLPLs in *Roquin^san/san^* mice differed fundamentally from those in normal mice in that *Roquin^san/san^* mice had an unusually high proportion of GR-1^+^ T cells as determined by CD3 expression. The majority of those cells were CD44^+^ and they included a subset of IL-17A secreting cells. A subset of CD8^+^ Gr-1^+^ cells express IFN-γ (panel **B** compared to panel **A**). Representative data from 2–3 normal and *Roquin^san/san^* mice.

The findings for siLPLs differed notably from that of siIELs in that only a small proportion of siLPLs were ICOS^+^ in both normal and *Roquin^san/san^* mice. However, a high proportion of siLPLs from *Roquin^san/san^* mice expressed Gr-1, which included a subset of Gr-1^+^ IL-17A^+^ cells (Fig. 7B). Although Gr-1 is most frequently associated with myeloid cells, a population of Gr-1^+^ CD8^+^ cells, some of which express IFN-γ, has been identified [10]. Because Gr-1^+^ siLPLs from *Roquin^san/san^* mice did not express F4/80 or CD11c antigens (not shown), and because the majority were CD3^+^, they were not macrophages or dendritic cells. Additionally, there also were more CD3^+^ siLPLs in *Roquin^san/san^* mice compared to normal mice (Fig. 7A and B), indicating that there had been an influx of T cells into the lamina propria of *Roquin^san/san^* mice.

We studied Gr-1^+^ siLPLs from *Roquin^san/san^* mice and normal mice with regard to expression of IFN-γ in the CD8^+^ population. These studies revealed significantly more IFN-γ synthesis by *Roquin^san/san^* mice compared to normal animals (Fig. 7B vs. A). Thus, siLPLs in *Roquin^san/san^* mice were a significant source of both IL-17A and IFN-γ, two potent pro-inflammatory cytokines.

### Cytokine dysregulation in the ileum of *Roquin^san/san^* mice

To understand how Roquin regulates the inflammatory response, we analyzed tissue sections from the ileum of *Roquin^san/san^* and normal mice for expression of 89 inflammation-associated genes as described in the Materials and Methods. Shown in Table 1, eleven analytes were up-regulated and ten were down-regulated in the ileum of 3*Roquin^san/san^* mice relative to 4 normal controls. Interestingly, in both the up-regulated and down-regulated groups, there were several analytes having significant chemotactic activities. This suggested that the alteration in the Roquin protein had a major impact on cytokine synthesis in the small intestine, and it implied that this Roquin disruption likely contributed to the phenotypic manifestation of the intestinal inflammatory response. Analyte synthesis by non-intestinal tissues was not determined.

**Table 1:** Changes in analyte levels in the ileum of three *Roquin^san/san^* mice relative to four normal control mice.

Upregulated		Down-regulated	
Analyte	Change	Analyte	Change
IL5ra	6.962	Tnfrsf1b	−4.917
CCL1	5.961	Actb	−4.401
CCL24	5.622	CXCL5	−3.118
PPC	2.578	Spp1	−2.826
IL13	2.356	CCL5	−2.781
IL1α	2.317	CCL4	−2.201
CCL20	2.313	CCR7	−2.146
CXCL13	2.193	IL1β	−2.082
CXCL1	2.187	IL6st	−2.068
CCL25	2.156	IFNγ	−2.004
IL4	2.159		

## Discussion

CD is a form of inflammatory bowel disease (IBD) that can affect any region of the alimentary tract and may be associated with extra-intestinal inflammatory conditions such as iritis, arthritis, pulmonary inflammation, and skin disorders [8], [11]–[13]. A number of mouse models exist for the study of IBD. These include cytokine knockouts such as IL-2^−/−^ and IL-10^−/−^ mice [14], [15], as well as models involving the transfer of effector T cells into immunodeficient mice [16], [17]. Inflammation in those models, however, is generally restricted to the colon. In contrast, there are few models of small intestinal inflammation that mimic CD. One of these is the SAMP1/yit mouse, which spontaneously develops ileitis by 20–30 weeks of age [18], [19]. Although that model has been useful for understanding various aspects of the inflammatory response in the ileum, it is problematic for studies into the biomolecular basis of disease because the underlying genetic changes responsible for the inflammatory phenotype have not been fully characterized. Small intestine inflammation also develops in mice in which TNF has been over-expressed, such as in TNF^ΔARE^ mice [20], [21], and in certain mouse strains following infection with *Toxoplasma gondii* [22].

The present study demonstrates that *Roquin^san/san^* mice are susceptible to the development of chronic small intestine inflammation. Although some *Roquin^san/san^* mice had modest intestinal pathology (11 mice had pathology scores ≤1.0), 12 mice had pathology scores ≥2.0. Only 2 of 23 *Roquin^san/san^* mice lacked pathology in the ileum. These findings indicate that the *Roquin^san/san^* mouse is a reliable animal system to focus studies on small intestine inflammation. Additionally and importantly, because the basis for the genetic defect in *Roquin^san/san^* mice is known, i.e., it is due to a mutation or deletion in the expression of the Roquin protein, it will be possible to design studies aimed at understanding the regulatory mechanisms by which Roquin controls intestinal inflammation. We recently identified miR-223 as one of the regulatory elements involved in controlling Roquin protein expression, and demonstrated that altering miR-223 expression affects Roquin expression [5]. Studies are currently underway to identify transcriptional factors that regulate the expression of Roquin, miR-223, and other Roquin-associated miRNAs.

*Roquin^gt/gt^* mice generated in our laboratory and used here may prove to be useful for studies into the involvement of Roquin in controlling intestinal inflammation. However, studies with *Roquin^gt/gt^* mice were limited by the fact that most mice died at birth. Analysis of 29 litters from heterozygous crosses revealed a ratio of 1.57 [wild type]: 4.30: [heterozygous]: 0.12 [homozygous gene trap], which is starkly different from the predicted simple Mendelian ratio of 1∶2∶1, and therefore suggested a role for Roquin in embryonic development, possibly in the lung [6]. To overcome this, we are currently using *Roquin^gt/gt^* bone marrow to immunologically reconstitute irradiated normal animals, which will circumvent the deleterious effects of Roquin on non-hematopoietic tissues while retaining its effect within the immune system. It also will be of interest to explore the extent to which inflammation in *Roquin^gt/gt^* mice can be exacerbated following exposure to pharmaceuticals such as piroxicam, a nonsteroidal anti-inflammatory drug that has been shown to accelerate the inflammatory response in IL-10^−/−^ mice [23]–[25], and in mice exposed to dextran sulfate sodium.

We found both similarities and differences between our *Roquin^gt/gt^* mice and the *Rc3h1^−/−^* mice that were produced by others [6]. Similar to mice in that study, *Roquin^gt/gt^* mice generated in our laboratory had a poor post-birth survival rate and most had a congenital caudal spine defect. However, unlike *Rc3h1^−/−^* mice which generally lacked autoimmune disease [6], *Roquin^gt/gt^* animals that lived to adulthood had a disease phenotype associated with the small intestine and liver that resembled that of *Roquin^san/san^* mice. Whether these differences have to do with how Roquin-deficient animals were generated, or whether the intestine is more prone to autoimmune disease, is unclear. The *Roquin^gt/gt^* mice used in our studies were created via random insertion of a β-geo gene trap vector into the *Roquin* locus. Mapping of *Roquin^gt/gt^* mice by 5′RACE positioned the gene trap vector between exon 1 and 2. This procedure normally results in complete gene inactivation, as appeared to be the case based on the lack of detectable Roquin protein in western blots. Bertossi et al [6] targeted exons 4–6 of the *Roquin* locus by flanking the exons with loxP sites to create *Rc3h1^−/−^* mice. Although western blot data from *Rc3h1^−/−^* mouse embryonic fibroblasts indicated a true null mutation, it is possible that this knockout strategy may leave truncated versions of Roquin produced from the remaining exons. The presence of a secondary band of lower molecular weight in western blots of TCR-β^+^ splenocytes in CD4-directed conditional Roquin knockout animals raises this possibility [6]. Regardless, our studies suggest that intestinal inflammation can develop in mice with either mutated Roquin or Roquin deletion.

The phenotypic profiles of MLN cells, siIELs, and siLPLs were revealing. MLN cells of *Roquin^san/san^* mice was characterized by an abundance of T cell activation markers, notably OX40, ICOS, and B220, as well as evidence for proliferating T cells, indicating replication of activated T cells. A second key feature of MLN T cells in *Roquin^san/san^* mice was the presence of CD44^hi^ CD62L^lo^ KLRG1^+^ SLECs. This is consistent with the finding of a population of SLECs in the spleen of *Rc3h1^−/−^* mice [6]. Similar findings linked to increased IFN-γ synthesis have been described for *Roquin^san/san^* mice [26]. Additional studies are planned in our laboratory using adoptive transfer experiments to trace the homing properties of the *Roquin^san/san^* SLECs to the gut, and using IL-7R expression to differentiate MLN SLECs from memory precursor effector cells (MPECs) [27]. Also of interest was the presence of increased numbers of FoxP3^+^ Tregs in the MLN of *Roquin^san/san^* mice. Although the significance of this is not yet clear, a recent study provided evidence for a functional role for ICOS^+^ FoxP3^+^ Tregs in the suppression of antigen-reactive T cells [28]. Additional analysis of that FoxP3^+^ T cell population in *Roquin^san/san^* mice may provide insight into how Roquin controls or fails to control autoimmunity.

siIELs from *Roquin^san/san^* mice were distinguished by the presence of ICOS^+^ CD44^+^ T cells; however, neither IL-17A nor IFN-γ were produced there to any appreciable degree. In contrast, ∼80% of siLPLs from *Roquin^san/san^* mice were Gr-1^+^ T cells, a significant proportion of which produced IL-17A. Also among siLPLs was a subset of GR-1^+^ CD8^+^ IFN-γ-producing cells, which may be a CD8^+^ memory T cell population [10]. Taken together, these findings suggest that the siLPLs are the likely leukocyte population responsible for inflammatory destruction of the intestine in *Roquin^san/san^* mice.

The inflammatory response in the ileum of *Roquin^san/san^* mice was complex as seen from the analyte studies done using ileum tissues, which revealed dysregulation in the expression of twenty-one immunologically-related analytes, eleven of which were over-expressed and ten were down-regulated. Collectively, the cytokines that were over-expressed would have a significant effect on the recruitment of monocytes and macrophages (CCL1), T cells, NK cells, and B cells (CCL24, CCL25, CCL20), dendritic cells (CCL1, CCL20, CCL25), and eosinophils and neutrophils (CCL24, CCL20) into the small intestine. Additionally, the presence of IL-1a, IL-4, IL-5ra, IL-13, and CXCL1 would have a strong effect on driving and sustaining an inflammatory response. Conversely, several analytes with proinflammatory activities were down-regulated in *Roquin^san/san^* mice. These included IL6st, IL1β, and IFNγ. Likewise, gene expression for analytes with strong chemotactic activities for T cells, eosinophils and basophils (CCL4 and CCL5), and memory T cells (CCL7) were down-regulated. Suppressed levels of CCL5 in the ileum of *Roquin^san/san^* mice differs from what has been reported for *Roquin^san/san^* mice [3]; however, the latter study used splenocytes not intestinal cells for analysis. The findings reported here point to a dynamic host process that probably reflects an effort to maintain a normal homeostatic response in the small intestine of *Roquin^san/san^* mice. How that balance is shifted in one direction or the other may ultimately determine the extent to which inflammation is mild or severe in those animals. It also will be of interest to determine the extent to which intestinal antigenic challenge following virus, bacteria, or parasite infection alters the inflammatory response locally and the extent to which this reflects the pathology seen in Crohn's disease, as recently reported for collagen-induced arthritis [29].

The findings reported here demonstrate that mice having a mutated Roquin protein, or animals in which the *Roquin* gene has been disrupted, are prone to the development of intestinal inflammation that is principally localized in the small intestine. Because of the relatively few animal models of CD pathophysiology in the small intestine, and because the primary gene-protein defect of the animals used here is known, it will be possible to pursue the genetic and molecular mechanisms that drive this process. These and subsequent studies, including experimental intervention designed to force Roquin expression in vivo, could be of considerable value for expanding our knowledge of factors that are causative in initiating and maintaining chronic intestinal inflammatory responses.

## Supporting Information

Figure S1 **Location of gene trap insertion into intron 1 of the Rc3h1 allele.** Black nucleotides are intron sequences. Green nucleotides are β-geo gene trap insert sequences.
(TIF)
