# Supplementary figures and images for: Small Intestine Inflammation in Roquin-Mutant and Roquin-Deficient Mice
Source: PLoS One. 2013 Feb 25;8(2):e56436. doi: 10.1371/journal.pone.0056436 (PMC3581552; doi:10.1371/journal.pone.0056436)

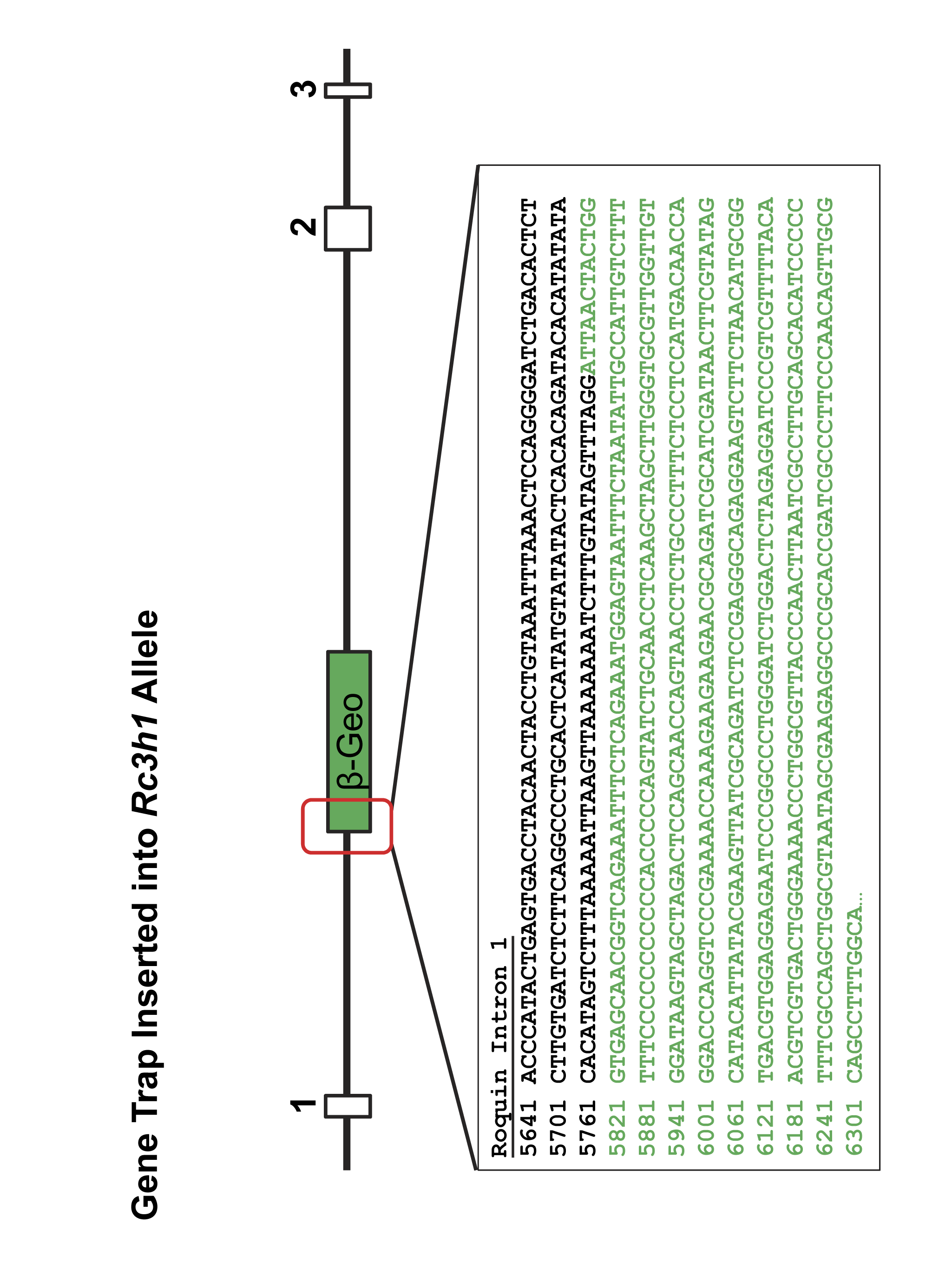

Supplement: Figure S1 — Location of gene trap insertion into intron 1 of the Rc3h1 allele. Black nucleotides are intron sequences. Green nucleotides are β-geo gene trap insert sequences. (TIF) [file pone.0056436.s001.tif]
